# High dose interleukin-2 (Aldesleukin) - expert consensus on best management practices-2014

**DOI:** 10.1186/s40425-014-0026-0

**Published:** 2014-09-16

**Authors:** Janice P Dutcher, Douglas J Schwartzentruber, Howard L Kaufman, Sanjiv S Agarwala, Ahmad A Tarhini, James N Lowder, Michael B Atkins

**Affiliations:** 1 Associate Director, Cancer Research Foundation, Chappaqua, NY USA; 2 Associate Director of Clinical Operations, Professor of Surgery, IU Simon Cancer Center, 550 N University Blvd, Indianapolis, 46202 IN USA; 3 Chief Surgical Officer and Associate Director for Clinical Science, Professor of Surgery, Rutgers Cancer Center Institute of New Jersey, 195 Little Albany Street, Room 2007, New Brunswick, 08901 NJ USA; 4 Chief of Medical Oncology and Professor of Medicine, St. Luke's Cancer Center, Bethlehem, 18015 PA USA; 5 Associate Professor of Medicine and Translational Science, University of Pittsburgh Cancer Institute, Suite 555, 5150 Centre Ave, Pittsburgh, 15232 PA USA; 6 Senior Medical Director, Prometheus Laboratories Inc, 9410 Carroll Park Drive, San Diego, 92121 CA USA; 7 Deputy Director, Professor of Medicine, Georgetown-Lombardi Comprehensive Cancer Center, 3970 Reservoir Rd NW, NRB-E501, Washington, 20057 DC USA

**Keywords:** Interleukin-2, Clinical management, Cytokines, Renal cell carcinoma, Melanoma, Treatment guidelines

## Abstract

Interleukin-2 (IL-2) was historically one of the few treatments for adults with stage IV solid tumors that could produce complete responses (CRs) that were often durable for decades without further therapy. The majority of complete responders with metastatic renal cell carcinoma (mRCC) and metastatic melanoma (mM) could probably be classified as "cures". Recent publications have suggested improved efficacy, perhaps due to improved patient selection based on a better understanding of clinical features predicting outcomes. Guidelines for clinical management were established from experience at the National Cancer Institute (NCI) and an affiliation of institutions known as the Cytokine Working Group (CWG), who were among the first to utilize HD IL-2 treatment outside of the NCI. As new centers have opened, further management variations have emerged based upon center-specific experience, to optimize administration of IL-2 and provide high quality care for patients at each individual site. Twenty years of evolution in differing environments has led to a plethora of clinical experience and effective management approaches. The goal of this review is to summarize the spectrum of HD IL-2 treatment approaches, describing various effective strategies that incorporate newer adjunctive treatments for managing the side effects of IL-2 in patients with mRCC and mM. The goal for IL-2 therapy is typically to administer the maximum number of doses of IL-2 without putting the patient at unacceptable risk for severe, irreversible toxicity. This review is based upon a consensus meeting and includes guidelines on pre-treatment screening, criteria for administration and withholding doses, and defines consensus criteria for safe administration and toxicity management. The somewhat heterogeneous best practices of 2014 will be compared and contrasted with the guidelines provided in 2001 and the package inserts from 1992 and 1998.

**Electronic supplementary material:**

The online version of this article (doi:10.1186/s40425-014-0026-0) contains supplementary material, which is available to authorized users.

## Introduction

Interleukin-2 (IL-2) was historically one of the few treatments for adults with stage IV solid tumors that could produce complete responses (CRs) that were often durable for decades without further therapy. The majority of complete responders with metastatic renal cell carcinoma (mRCC) and metastatic melanoma (mM) could probably be classified as "cures". Despite this remarkable response quality in a small minority of patients, the requirement for hospitalization at expert immunotherapy centers to manage the side effects of high dose IL-2 (HD IL-2) administration has limited its utilization. Early studies evaluated high dose IL-2 in comparison with lower doses both in mRCC and mM in an effort to minimize toxicity. However the data demonstrated superiority of the high dose (HD) regimens in both diseases [1]-[4]. Faced with the task of administering of HD IL-2, guidelines for clinical management were established from experience at the National Cancer Institute (NCI) [5]-[7]. An affiliation of institutions known as the Cytokine Working Group (CWG), who were among the first to utilize HD IL-2 treatment outside of the NCI, led the implementation of management strategies in new settings [8]. Currently there are more than 60 centers in North America administering HD IL-2 for mRCC and mM. As new centers have opened, further management variations have emerged, based upon center-specific experience, to optimize administration of IL-2 and provide high quality care for patients at each individual site. Twenty years of evolution in differing environments has led to a plethora of clinical experience and effective management approaches.

Recent publications have suggested improved efficacy, perhaps due to improved patient selection based on a better understanding of clinical features predicting outcomes [9]-[11]. At the same time, the number of doses of IL-2 being administered per course has decreased, reducing toxicity without an apparent impact on efficacy [1],[2],[7]-[10],[12]-[14]. These trials have stipulated the standard dose and schedule of HD IL-2, but this delivery and management could vary according to each site's tolerance for specific toxicities and approach to managing them. Virtually no information exists in the literature regarding the specifics of administration practices or median doses per cycle given at major HD IL-2 centers.

In recent years, new therapies have been approved in both mRCC and mM. Prior treatment with these new agents may influence the eligibility of patients for HD IL-2, modify their response, or change the management strategies of HD IL-2. In many instances, little information exists in the literature regarding the integration of HD IL-2 therapy with other treatments and more guidance would be desirable.

The goal of this review is to summarize the spectrum of HD IL-2 treatment approaches, describing various effective strategies that incorporate newer adjunctive treatments for managing the side effects of IL-2 in patients with mRCC and mM. The goal for IL-2 therapy is typically to administer the maximum number of doses of IL-2 without putting the patient at unacceptable risk for severe, irreversible toxicity. This review is based upon a consensus meeting and includes guidelines on pre-treatment screening, criteria for administration and withholding doses, and defines consensus criteria for safe administration and toxicity management. The somewhat heterogeneous best practices of 2014 will be compared and contrasted with the guidelines provided in 2001 and the package inserts from 1992 and 1998.

### Early physiology investigations of high dose IL-2 therapy

In the 1980's and 1990's, clinical trials evaluating the efficacy of HD IL-2 yielded a great deal of information on the physiologic effects of exogenous, HD recombinant IL-2 administration in humans [15]-[27]. Early clinical studies employed IL-2 often in combination with adoptively transferred lymphocytes referred to as Lymphokine Activated Killer (LAK) cells. In these studies, it was not possible to separate the contribution of each modality in terms of clinical response and emergence of treatment-related adverse events. Further complicating the picture was the fact that the dose and schedule of IL-2 administration often varied from study to study. Although the specific molecular and cellular mechanisms of IL-2-mediated response and toxicity are not completely defined, IL-2 is known to result in a cascade of cytokines released at supraphysiologic levels in the body from IL-2-activated cells. This can result in a well-described capillary leak syndrome and eventual end-organ dysfunction [15]-[27]. The hallmarks of this "cytokine storm", are remarkably similar to the shock syndrome associated with bacterial septicemia, and include: sustained hypotension, tachycardia and increased vascular permeability. In addition, high serum cytokine levels appear to be directly or indirectly toxic to the cells of multiple organs [28],[29]. Early animal and human studies suggested a dose-response effect on both efficacy and toxicity [15]-[17]. Thus most of the early literature described alternative methods of managing these patients than those used for sepsis, as the desirable therapeutic effects were thought to be linked to the toxicity (5-8,24-27) and the inciting factor (IL-2 administration) was within the physician's control.

Studies of cardiovascular physiology evaluated the universal hypotension associated with HD IL-2 administration and demonstrated high cardiac output and reduced peripheral vascular resistance (42% mean decrease) and a decline in mean cardiac ejection fraction from between 58% to 52% [24]-[26]. Six of ten patients in one study in which patients received HD IL-2 alone, had reductions in their LVEF of 5% (absolute number) or greater. In a smaller study where patients were also administered LAK cells, mean ejection fraction fell from 58 to 36%. An increase in ejection fraction would be expected with the increased inotropy and decreased afterload associated with hypotension. These studies suggested that HD IL-2 administration and the associated high serum levels of other cytokines, produced toxic effects on the myocardium which are worsened by the co-administration of LAK cells. Based upon temporal correlation of levels, TNF-α, may be the central cytokine in this response [27]. Both studies demonstrated complete recovery of all parameters, even at 14 days, following IL-2 administration cessation.

Renal dysfunction was virtually always reversible, and was evaluated as being both pre-renal and renal in origin [30]-[32]. The sustained hypotension certainly reduces renal blood flow and results in a pre-renal dysfunction characterized by high aldosterone and renin, and high tubular recovery of sodium. Patients receiving HD IL-2 may become severely oliguric and even anuric, but rarely manifest acute tubular necrosis, as might be expected with purely hypotensive effects. The falling BUN:creatinine ratio and decreased filtration fraction speaks to an intrinsic renal defect probably mediated by a downstream cytokine. The minimal proteinuria suggests that the increased vascular permeability seen in the rest of the body is not active at the glomerular level. The rapid and complete recovery suggests that no real structural damage occurs during IL-2 treatment.

Infections were a frequent cause of adverse events and even death in early trials of HD IL-2. The majority of these infections were related to intravenous catheters, largely with gram positive bacteria. These were attributed to the use of central venous multiport catheters necessary to administer high rates of fluids or vasopressor medication [33],[34]. Aseptically-placed catheters developed infections much earlier than would have been predicted. A granulocyte migration defect observed in patients receiving HD IL-2 was postulated to have permitted local infections to become systemic [33]. The use of prophylactic antibiotics, fresh placement and removal of intravenous catheters with each cycle of treatment have largely addressed this issue [6],[7].

Making standard recommendations regarding the amount of HD IL-2 administration is complicated, as IL-2 is typically delivered as an intravenous bolus with a fixed dose based on individual patient weight (typically either 600,000 or 720,000 Units/kg) and then that dose delivered every 8 hours up to a maximum of 14-15 doses subject to toxicity. Most patients receive one course of treatment that consists of two cycles separated by a 9-14 day holiday before re-staging. The first cycle of each course has been associated with more doses than the second in the vast majority of individuals. Comparing total dosing is also confounded by the dropout of non-responders after the first course. Studies do not report dosing in any routine fashion making comparisons difficult. Evolution of management during the first decade of treatment with HD IL-2 at the NCI resulted in a 58% reduction in number of doses in the first cycle of IL-2, with a concomitant significant reduction in grade 3 or 4 toxicity (reduction in diarrhea by 80%, reduction in neurotoxicity by 50%, reduction in serious pulmonary toxicity by 75%, and reduction in mortality to zero) [7]. An average of 8 x 720,000 IU/kg (equivalent to about 9 600,000 IU doses) was established for the first cycle at the NCI. Although no statistically significant difference in the CR rate or response duration for either mRCC or mM patients was associated with these changes some early studies showed correlation of response with the number of doses [1],[7],[35]. Studies in the succeeding decades by the CWG, have shown a modest decrease in the number of doses administered during the first course, from a mean of 22 in the 1980's-early 1990's, [3] to a mean of 19 in studies published in early 2000's, [2] to 16-20 doses in most recent studies [9],[10],[13]. Variations in response rate are within the bounds expected from relatively small trials, [9],[10],[13] and an acceptable level of grade 4 toxicity has been achieved without sacrificing efficacy [9],[10],[13]. All recent studies demonstrate rapid reversibility of all expected IL-2 related toxicities.

## Review: Where is high dose IL-2 management today?

Screening of patients has changed as medical practice and technology has changed. Management of cardiovascular disease is dramatically different now than 25 years ago. Physiologic age is recognized as more important than chronological age. Prior therapy in patients encompasses many more options, some of which may influence eligibility for and tolerability of HD IL-2. Current approaches to toxicity management are more variable, but largely with the same technology. The same fluids and vasopressors are used for the management of hypotension. Advances in anti-nausea agents have improved this aspect of care. The site in the hospital where IL-2 is administered (ICU vs step down unit, vs regular inpatient ward) and the aggressiveness in the number of doses per cycle are highly variable from site to site. Different parameters for screening and management are utilized according the experience and comfort of the site personnel. The tables in this paper show recommended ranges for continuous variables and variations in management beyond the typical approach.

### Screening (Table 1)

**Table 1:** **Pre screening recommendations for high dose IL-2 therapy**

	Package insert guidelines	Current best practices
**Disease**	**MM or mRCC**	**MM: cutaneous better than mucosal/ocular mRCC: clear cell/mixed histology**
PS	0-1	0-1
Cardiac function	**All patients**	**Under 40-50 without history**
	Thallium stress test-normal	Selective testing only
		**Over 40-50 or prior history**
		EKG stress test-normal (off beta blockers)
		**CAD corrected > 6-12 months**
		EKG stress test, EF, motility-normal
		Cardiology consult
		**Prior TKI**
		3 month gap if possible
		EKG stress test, EF, motility-normal
		**Abnormal stress test**
		Cardiology consultation
Pulmonary function	**All patients**	**Under 40-50 without history**
	PFTs with ABGs	Selective testing only
		**Over 40-50 or with history of smoking or pulmonary disease**
		FEV1 ≥ 75% predicted
Brain metastasis	No untreated CNS metastasis	**Negative MRI**
		**Positive MRI**
		Treated, asymptomatic, off steroids
Infection	No active infections	No active infections
Renal function	**All patients**	**Serum creatinine ≤ 1.5 mg/dL**
	Serum creatinine ≤ 1.5 mg/dL	**Serum creatinine 1.5-2.0 mg/dL**
		Creatinine clearance > 60 ml/min
Laboratory	**All patients**	Bilirubin ≤ 2.0 mg/dL
	Values within normal limits	Platelet count > 100,000
		Hemoglobin ≥ 9.0 g/100 ml
		ANC ≥ 1500/mm^3^
		SGOT < 3x ULN
		Thyroid function normal
Other	**No steroids, autoimmune disease, allografts**	

Substantial changes in the supportive and interventional management of disease as well as diagnostic technology have occurred during the past 3 decades. In addition, new therapies have become available whose chronic toxicity may impact a patient's ability to receive HD IL-2 subsequently. HD IL-2 package insert instructions are based upon literature and experience generated during the 1980's and 1990's. What was viewed as an absolute contraindication to therapy or a screening necessity in the past may not be sensible in today's environment. Nonetheless, careful attention to screening of patients prior to IL-2 treatment remains critical to successful and safe administration of this treatment. Patients with eligibility concerns should always be evaluated by the relevant subspecialist prior to therapy and ideally followed by that physician during therapy. Less experienced sites, however, should always err on the side of more conservative screening.

The single most important parameter for screening is a patient's functional - or performance - status, as measured by the ECOG or Karnofsky score as this predicts likelihood of response, as well as, tolerance of the therapy. Only patients with intact (ECOG = 0 or K = 100) or minimally impaired (ECOG = 1 or K = 80-90) function are candidates for HD IL-2. Functionality represents an excellent sum of the patient's total organ and physical reserve which will be severely taxed during therapy. Secondly, the histologic and molecular subtypes of RCC and melanoma have led to more stringent selection by histology (see variations on disease type in Table 1) based on likelihood of response to IL-2 therapy [36].

#### Cardiovascular

Although cardiac toxicity remains a major concern in HD IL2 treatment, the baseline risk has diminished in the general population in recent decades. The reduction in cigarette smoking, aggressive prophylaxis with statin drugs and management of hypertension and cardiovascular disease has significantly reduced the incidence of occult cardiac disease. The use of coronary artery bypass grafts and stents has corrected existing CAD with preservation of cardiac functional reserve. Careful attention to family history, risk factors and lifestyle is important in determining the extent of cardiac risk and degree of screening needed in preparation for HDIL2 treatment. Although the package insert mandates thallium screening in all patients, most sites follow different approaches, as the thallium stress test produces many false positive results. Patients below the age of 40-50 without relevant history or risk factors generally may not require extensive screening. Additionally, patients with prior history of coronary artery disease which has been corrected may be considered for treatment if this occurred at least 6-12 months in the past, but should be tested [37],[38]. The extensive use of VEGF targeted tyrosine kinase inhibitors in mRCC is an important historical issue. These agents can be cardiotoxic and can produce reductions in left ventricle ejection fraction, particularly if treatment duration has exceeded one year. Ideally, patients should be off anti-vascular endothelial growth factor receptor tyrosine kinase inhibitors (anti-VEGFR TKIs) for 3 months before HD IL-2 [39]. Careful and complete screening is mandatory in these patients. An EKG exercise treadmill stress testing is the standard modality for testing patients. Ultrasound assessment of ejection fraction and myocardial wall activity are valuable parts of the stress test and may be employed in patients with risk factors. Ideally, stress testing should be done with the patient off of beta blockers, as they will be discontinued during HD IL-2 therapy. Dipyridamole (Persantin®) stress testing may be used in patients unable to effectively use a treadmill. If the stress test is abnormal, a cardiologist should be consulted, and ultimately determine both the need for and the path of further evaluation to help make a final decision about eligibility.

#### Pulmonary

Due to pulmonary fluid overload and the demand on the myocardium caused by hypotension, adequate oxygenation of the blood may be an issue. Smoking history and careful attention to diseases compromising pulmonary function are important signs of reduced pulmonary reserve. Restrictive lung disease may be debilitating during the stress of therapy. Patients without relevant history below the age of 40-50 do not require screening unless there are risk factors or signs or symptoms of underlying pulmonary dysfunction. An FEV1 > 75% of predicted is adequate for predicting IL-2 tolerability. Any other tests should be ordered at the discretion of a pulmonary consultant. Pulmonary function studies may also be useful for defining the patient's baseline pulmonary function, which can help in management of changes observed during treatment.

#### Brain metastasis

Good quality magnetic resonance imaging of the brain should be obtained prior to starting HD IL-2. Patients with brain metastases were often considered not to be candidates, however, with the advent of stereotactic radiotherapy, an asymptomatic patient with adequately treated isolated brain metastases and off systemic corticosteroids can be considered for treatment. Similarly, patients with small untreated brain metastases that are limited in number and causing minimal or no edema may be considered for IL-2 treatment [40].

#### Effusions

Patients with large pleural effusions or large amounts of abdominal ascites are not good candidates for HD IL-2. The fluid shifts experienced during IL-2 treatment generally results in expansion of existing fluid collections and will likely result in exacerbation of symptoms that will preclude effective therapy with IL-2.

#### Infections

All patients should be carefully screened for infection prior to IL-2 treatment and any active infection should be fully treated and the patient should be off antibiotics prior to starting a cycle of HD IL-2. This includes clearance of C. difficile if that has been identified. In addition, tumor related impingement on structures which might lead to closed space or obstructive infections should be carefully evaluated and corrected if necessary.

#### Other

Most other parameters are assessed according to laboratory values which are listed in Table 1.

In the hands of experienced therapy teams, treatment can be customized with a sense of the patient's physiologic reserve. This will help to guide the setting of the target blood pressure, the need for specialist support and the threshold for stopping and holding doses of IL-2.

### Site of administration unit

HD IL-2 administration requires trained experienced staff and an inpatient location where cardiac and O2 saturation monitoring are available and vasopressor agents such as dopamine and phenylephrine can be administered according to hospital policy. In addition, a low nurse to patient ratio is necessary. These requirements are fulfilled in specialized oncology floors, transplantation units, intensive care step down units or intensive care units in various institutions. Novice sites might be advised to treat up to 10 patients in an intensive care setting until they are comfortable, then move to a less intensive and expensive setting, provided there is sufficient clinical support. If intensive care nurses or physicians are involved, they should be carefully trained in how to care for the HD IL2 patients, as management is often counterintuitive to the management of the usual intensive care patient. Transfer of patients from one setting to another is to be avoided if at all possible as nursing continuity is critical [41]. If patients being managed outside the ICU develop problems that cannot be managed safely in the current location (e.g. cardiac arrhythmia, respiratory failure, bacterial sepsis) such patients should be promptly transported to an ICU and further IL-2 treatment stopped.

### Overall schedule and dose

Most centers continue to utilize the original empirically defined NCI schedule of one week on (cycle 1), one week off, one week on (cycle 2), defined as one "course" of treatment. The inter-cycle time may be extended by another week based upon patient recovery or site preference. The disease response, assessed at 6-12 weeks from therapy initiation, after a full course of therapy helps determine the value of administering another course. Any response is generally viewed as a reason to give another course. If stable disease is observed, delay of an additional 4-6 weeks or longer followed by reassessment may inform the decision for further dosing. Continued disease stability over months in a patient with clearly progressive disease before therapy is frequently, although not always, viewed as reason to continue treating. Of note, in one study, 90% of patients that achieved a response to treatment, did so after one course of IL-2 [42]. In light of these observations, decisions about additional courses of IL-2 should be individualized based on toxicity of prior therapy, extent of residual disease and availability of alternative treatment options. The interval between courses may vary considerably for a variety of other reasons including patient recovery and logistics. The standard dose of HD IL-2 remains 600,000 IU/kg (based on initial studies), although higher and lower approaches have been described in the literature [1]-[13],[43]. The NCI and some sites use 720,000 IU/kg [1]. For the most part, doses are given as a short bolus infusion (over 15 minutes) every 8 hours, over the course of 5 days. Toxicities generally occur over the 6 hours following a bolus dose, abating before the next scheduled dose. If recovery from the previous dose is not noted at 8 hours, some centers skip a dose and resume the 8 hour schedule. Some sites extend the time between doses to 12 hour intervals at this juncture. Individual *dose reductions should not be used.* Treatment extends for 5 days, stopping when the 14th planned dose would have been administered (end of day 5) regardless of the number of doses administered. Treatment is typically concluded earlier when adverse events occur that make further dosing intolerable, thus only a small minority of patients receive all 14 doses. The IL-2 treating physician will decide when to hold or stop dosing during a cycle based upon criteria to be discussed later in this paper. The current total mean number of doses administered is in the range of 16-20 of the 28 maximum doses in the two cycles of treatment.

Some institutions have adopted schedules that continue to use the standard amount of IL-2 per dose but offer variations in dosing interval and number of dosing cycles [12],[44]. These approaches indicate that interest remains in optimizing IL-2 therapy in ways which maintain efficacy but improve the safety profile for patients. Little data exists on the dose response curve, however, the response rate between 600,000 or 720,000 IU/kg (HD) and 72,000 IU/kg (moderate dose) is significantly different [1].

### Initial admission and management - standing orders (Additional file 1)

Most centers have a set of routine IL-2 orders that establish the basic procedural approach of the local IL-2 team. Although these vary considerably from site to site, they are strictly adhered to within a site, in order to maintain continuity among nursing shifts and facilitate clinical decision making. Standard protocols for maintaining intravenous access catheters, intravenous fluids, prophylactic-and as needed (PRN) medications for symptom control, monitoring parameters, and daily laboratory evaluations, guide the daily management of patients. Reactive management of expected complications, such as hypotension, decline in oxygen saturation, tachycardia, and alterations mental status should also be listed. Threshold values for various monitoring parameters should be listed which require contacting the physician. Standing order specifics are imbedded in Table 2. Additional file 1 provides an example of standing orders. These parameters take into consideration the standards in each clinical unit, the type of monitoring available and the philosophical approach of the team. The frequency of blood testing is usually once daily, with additional testing as needed and directed by the IL-2 physician. Patients are admitted to the hospital the evening before or the morning of the first IL-2 infusion. The most common dosing schedule is 8 AM, 4 AM and midnight often with the first dose being administered at 4 AM on day 1.

**Table 2:** **Clinical management recommendations for HD IL-2 therapy**

Issue	Considerations	Management
**Venous access**	Central line (for possible vasopressors)	**Typical**
	Double or triple lumen	PICC line placement
	Power inject and large volume capacity	Remove temporary lines at end of cycle
	Minimize catheter associated infection	**Variations**
		Broviac/Hickman catheter
		Subclavian/IJ catheter
**IV fluids**	Maintenance of volume with CLS	**Typical**
	Boluses for blood pressure support	D5NS or D5LR 10 ml - 125 ml/hr
	Administration of drugs	PRN KCL, HCO3, Mg replacement
	Replacement of electrolytes	**Variations**
	IL-2 only compatible with D5W	D5W, NS, 0.45% NaCl
**Infections**	No active infections	**Typical**
	Prevention	Gram + prophylactic antibiotic
	IV catheter likeliest source	**Variations**
	Avoid unnecessary in-dwelling catheters	Expanded coverage per hospital
**Chills/rigors**	Chills and rigors occur 1-2 hrs after IL-2	**Fever-Typical**
		Prophylaxis
**Fever**	Fever is common 2-4 hrs after IL-2	Acetaminophen 650 mg 30 min pre-dose, q 4-6 hrs and prn
		Indomethacin 25 mg q 6-8 hrs
**Constitutional symptoms**	Muscle joint aches continuous and progressive during IL-2 treatment	**Fever-Variation**
		Naproxen
		Ibuprofen
		**Chills-Typical**
		Meperidine 25 mg IV q 15 m prn
		Morphine 2-4 mg IV q 15 m prn
**Nausea/vomiting**	Episodic occurrence throughout therapy	**Typical- Prophylaxis**
	Nausea > vomiting	Ondansetron 0.15 mg/kg q 8 hrs
		**Variations**
		Granisetron 1 mg daily
		Ondansetron at longer interval
		Compazine 10 mg po q 6 hrs
		Use of antinausea agents prn
**Epigastric distress**	Gastritis induced by stress, medications	**Typical**
		H2 blocker prophylaxis
		**Variation**
		PPI prophylaxis
**Mucositis/stomatitis**	Progressive with continued treatment	**Typical**
		No prophylaxis
		Oncology mouthwash
**Diarrhea**	Can be profuse and increases with therapy	**Typical**
	5HT-3 antagonist anti-emetic prophylaxis	Imodium
	may have positively impacted	Lomotil
		Narcotic
		Break between IL-2 doses
		**Variations**
		5HT-3 antagonist prophylaxis
**Patient monitoring**	I & O, Weight	Per shift and daily
	Blood pressure, pulse, respirations, temp	Q 2-4 hrs
	Blood work	Daily
	EKG	Continuous cardiac monitoring
	O2 Saturation	Q 2-4 hrs
	Mental status examination	Q 8 hrs
		**Increase frequency as needed**
**Aldesleukin/Interleukin-2 dose and administration**	IL-2 incompatible with salt solutions.	**Typical:** 600,000 IU/kg infused over 15 minutes Q 8 hrs up to 14 doses.
	Dissolve in sterile water for injection Dilute into 50 cc D5W	**Variations:** 720,000 IU/kg Q 8 hrs Q 12 hrs < 14 maximum doses
	Stop infusion, flush IV tubing with 50 cc D5W before and after each dose.	
**Hypotension**	Maintain systolic BP 80-90 mm hg	Fluid boluses, 250-500 ml NS
	Blood pressure nadirs 4-6 hrs after each dose with diminished recovery with cumulative dosing	2xday
		Increase maintenance fluid rate
		Phenylephrine 0.1-4.0 mcg/kg/min
		Hold next dose
	Prior to each dose anticipate ability to respond to next nadir	DC IL-2
		**Variations:**
		Dopamine 1-6 ug/kg/min
	Progressive refractoriness to support measures	Pressors with minimal fluids
		Fluids without pressors
**Cardiac arrhythmias**	**Sinus tachycardia**	Manage BP and fever
	Common and progresses over a cycle	
	Peaks 2-4 hrs after dose with fever and hypotension	
	Must resolve prior to next dose	
	**Supraventricular tachycardia, atrial fibrillation**	Medical Conversion
	Less common	Cardizem as needed
	**Atrial fibrillation**	Digoxin
	**Ventricular tachycardia**	Medical Conversion
		Acute treatment
		Discontinue IL-2
**Renal function**		**Typical**
	**Oliguria**	Output less than 50-100 cc/8 hrs Fluid bolus, if no improvement next shift hold IL-2 dose
	**Rising creatinine**	Creatinine >3-4
		Stop NSAIDS and nephrotoxic antiobiotics
	Urine output and creatinine resolve after discontinuation of IL-2	Hold overnight dose
		If am creatinine improved continue
	If only one kidney always consider obstruction of ureter	**Variations**
		Dopamine 1-6 mcg/kg/min
		Furosemide
**Pulmonary**	Tachypnea/Dyspnea	**Typical**
	Diagnose etiology and treat	Oxygen 2-4 L nasal cannula, increasing up to 35% rebreather
	Hypoxic causes-Fluid overload, capillary leak, bronchospasm	Reassurance or sedative for anxiety, treat bronchospasm or acidosis if appropriate
	Non hypoxic causes	Hold IL-2 dose if O_2_sat < 95%
	Anxiety, fever, acidosis	
	**Maintain O**_**2**_ **sat > 92-5%**	**Variation**
		Furosemide
		Bronchodilators
		Monitor bicarbonate
**Peripheral edema**	Expect to gain 5-10% body weight	Elevation, compression, limit fluid support in subsequent cycles
	Treat edema symptomatically	Diuretics upon conclusion of IL-2 dosing are not necessary but may speed process
	Entrapment of peripheral nerves in upper extremity may need therapy	Treat peripheral nerve pain
**Neurotoxicity**		**Typical**
	Protean manifestations	Formal neuro checks
	Gradual onset with sudden worsening near end of cycle	Enlist family evaluation
	May persist after cessation of therapy	Lorazepam and Haloperidol
	Delusions, Visual hallucinations	Hold IL-2 liberally for suspected neurotoxicity
		Warn patient of vivid dreams after discharge
**Dermatologic**		**Typical**
	Rash, erythema, dry desquamation	Emollient lotions and creams Oatmeal bath
	Pruritus	Antihistamines
	Moist dermatitis	Hold IL-2 dose
		**Variations**
		Crisco
		Gabapentin
		Naloxone
		Narcotics
		**Nonalcohol, no steroid topicals**
**Metabolic**	Hypomagnesemia, hypocalcemia (but low albumin - so corrected may be WNL), Hypokalemia-	Daily electrolyte panels
	Acidosis due to diarrhea, hypoperfusion	Correct electrolytes cautiously prn
	Hypothyroidism a slow onset problem	Magnesium and HCO3, particularly if diarrhea a problem
		HCO3 < 18 meq/L hold dose of IL-2
		Check TSH at beginning of cycle
		RL as support fluids may decrease need for HCO3
**Hepatic**	↑Bilirubin (up to 10)	Monitor daily
	↓Albumin (down to 1.8)	No intervention except if SGOT SGPT are >5x
	↑Hepatic aminotransferases	Resolves spontaneously
		Stop acetaminophen if bilirubin > 5
**Hematologic**	↓Platelets	Transfuse platelets if < 20 K
	Lymphs ↓during IL-2, ↑post therapy	Other abnormalities require no intervention
	Eosinophils progressively ↑with several cycles	Significant anemia needs evaluation for cause
**Endocrine**	Hypothyroidism - slow onset after completion of treatment	Check TFTs at beginning of cycle and monitor TFTs with subsequent visits
	Requires serial monitoring	

### Toxicity management by organ system - typical management and variations (Table 2)

The most important skill in managing an HD IL-2 patient is to understand when to withhold the next dose. Acute inter-dose exacerbations peak around 4-6 hours following each dose. Before another dose is given, patients should be approaching or have returned to their target baselines for heart rate, oxygen saturation and blood pressure. There should be capacity to provide increased blood pressure and oxygenation support during the inter-dose exacerbation which will follow administration of the next dose. Toxicities are generally cumulative, worsening and becoming refractory to management with successive doses during the week. Preparing patients and family to expect symptoms will alleviate their fears and enlist their support.

#### Fever/chills

These are the first side effects encountered by patients and can be quite disconcerting. Almost all patients have fever with at least the first IL-2 dose, despite premedication, usually within 30-60 minutes after the dose. Over the week, these symptoms generally lessen with subsequent doses. Both acetaminophen and non-steroidal anti-inflammatory agents (NSAIDS) are administered prophylactically and as needed for fever. Continuous NSAIDS are not a problem despite a rising creatinine, in view of the universal reversibility of the renal adverse events seen with IL-2. However, holding or withdrawing NSAIDS later in the course when the creatinine rises above 3-4 mg/dL is prudent.

Often fever comes after an episode of severe chills or rigors. Significant rigors are effectively managed by giving parenteral opioids immediately at the onset of the rigor. Although meperidine is the most commonly used agent, morphine is also used and may be less emetogenic. Fevers and chills which occur outside the 2-6 hour window after a dose of IL-2 must be considered as potentially due to infection and evaluated accordingly.

#### Hypotension

This is the most common and important toxicity of HD IL-2 treatment (70% of patients in early studies), reflecting capillary leak, decreased peripheral vascular resistance with high cardiac output, characteristic of systemic inflammatory response syndrome (SIRS). A variety of causes of gap junctional instability in endothelial cells have been postulated as the underlying mechanism for the leak. The relationship of capillary leak to the hypotension is uncertain as changes in peripheral vascular resistance may produce decreases in blood pressure even in the absence of capillary leak. Hypotension is largely unavoidable and may be viewed as a clinic-dynamic parameter for drug administration. Standing orders should specify a new baseline tolerable systolic blood pressure of at least 20 points lower than baseline was prior to initiating IL-2. In addition, a wide pulse pressure should be expected from a decrease in diastolic pressure. The baseline values should be customized for each patient taking into account the individual's cardiac status and prior exposure to tyrosine kinase inhibitors directed at the vascular endothelial growth factor pathway. During treatment this level of hypotension becomes the new baseline that is the goal for the entire dosing period. Typically, the acceptable target systolic pressure is about 80-90 mmHg for patients without cardiac risk factors, but may be adjusted upward in patients who are at higher risk of coronary or cerebral artery disease. Management of blood pressure also requires maintaining an acceptable heart rate which will be discussed later.

The management of hypotension also appears to have the broadest spectrum of practice among centers. The approaches hinge on a preference for using fluids versus vasopressors to maintain blood pressure (BP). Some centers rely solely on large volumes of saline, lactated ringers or hetastarch to maintain blood pressure with the expectation that the patient will gain at least a kilogram or more per day, with a total weight gain of 5-10% of their body weight during the week of administration. The relative values of colloids vs. crystalloids have been studied extensively in intensive care situations with no obvious advantages, except for cost [45]. Fluids are administered as continuous infusion or as boluses, in response to hypotensive episodes. Initial maintenance rates vary between of 5-125 ml/hour. Bolus sizes vary from 100 ml to 500 ml. Since patients can experience flash pulmonary edema, fluids should be administered cautiously during active IL-2 treatment. Other centers will start a vasopressor, particularly dopamine or phenylephrine, immediately and more sparingly employ maintenance and bolus fluids [46]. Patients with a history of hypertension admitted for HD IL-2 therapy should have outpatient antihypertensive medications discontinued immediately prior to IL-2 therapy.

### Examples

**Center A** routinely administers HD IL-2 on the oncology unit. They utilize maintenance fluids of 75-100 ml/hour. For hypotension, patients are initially given 250-500 ml of saline, LR or hetastarch with a second bolus of 250 ml allowed to raise the BP to the target systolic pressure. Dopamine is used on the oncology unit if dose-limiting renal dysfunction is experienced, but no other vasopressors, and it is used at renal doses (1-5 ug/kg/min). If a patient develops significant tachycardia while on dopamine (greater than 120- 130/min sustained), they hold IL-2, correct intravascular hypovolemia and allow the patient to recover. If the patient becomes fluid overloaded with respiratory symptoms, they hold or stop IL-2, and use diuretics as necessary, prior to respiratory compromise. The goal is to administer up to14 doses/cycle if dosing criteria are met before each dose.

**Figure 1:**
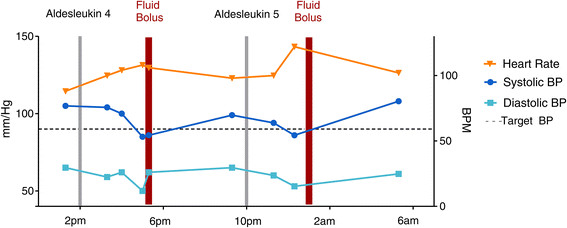
**Timing of the administration of the 4th and 5th IV boluses of IL-2 is plotted versus systolic and diastolic blood pressure and heart rate.** This patient shows characteristic exacerbation of hypertension 3-4 hours after infusion responsive to fluid boluses.

Figure 1 shows a patient with a target systolic BP of 90 experiencing BP reductions with tachycardia 3-4 hours after doses 5 and 6 of IL-2. These episodes responded promptly to a 500 ml bolus of saline.

**Center B** treats patients in the ICU in order to maintain a lower nurse to patient ratio and permit the use of vasopressors. Patients are given maintenance fluids of 10 ml/hour unless they have excessive nausea/vomiting or diarrhea, in which case it is raised to 30-50 ml/hour. Volume boluses are used as previously described for episodes of hypotension. If this does not reverse the hypotension, then patients are started on phenylephrine at 50 micrograms/minute (0.1-2.0 ug/kg/min), titrated as necessary to maintain BP, with a preset maximum dose level individualized per patient cardiac risk factors between doses. The phenylephrine must be tapered back to baseline of 50 micrograms/minute or less before another IL-2 dose is given. With subsequent IL-2 doses, up to 2 fluid boluses per 8 hours may be administered for hypotension if there are no signs of pulmonary compromise or excessive (>5% baseline weight) fluid overload, but vasopressors are the mainstay of managing hypotension in this setting. The need for a 3rd bolus in 24 hours generally requires holding the next dose of IL-2. Some patients become refractory to phenylephrine and require dopamine or norepinephrine (Levophed™). Even with this regimen, patients usually gain 10-20 pounds of water weight during a 5 day course of treatment.

**Figure 2:**
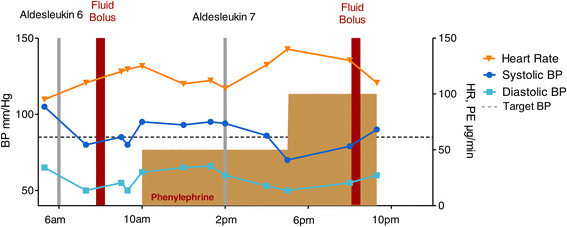
**Timing of the administration of the 6th and 7th IV boluses of IL-2 is plotted versus systolic and diastolic blood pressure and heart rate.** This patient is unresponsive to a fluid bolus and phenylephrine is begun at 50 mcg per minute with good effects permitting administration of the 7th dose. The resulting hypotension and tachycardia are treated with an increase in the phenylephrine dose and another bolus of fluid. The late night dose will likely be held.

Figure 2 shows the course of a patient responding minimally to a bolus of saline after suffering hypotension and tachycardia after dose 6 of IL-2. Phenylephrine is started at 50 mcg/kg/min with return of BP and HR to acceptable levels. After the next dose hypotension and tachycardia recur and respond minimally to an increase in phenylephrine dose and better to an additional bolus of saline.

Experienced centers use phenylephrine and dopamine on an oncology or stem cell transplant unit, which has the nursing staff trained to support such use and also have cardiac monitoring capability. Their management practice is similar to that described in Center B. In any scenario, the blood pressure/heart rate must recover to a predefined baseline prior to administering the next dose of IL2.

#### Cardiac arrhythmias

There is fairly uniform practice with respect to managing cardiac arrhythmias with serious arrhythmias being uncommon. Sinus tachycardia is to be expected and a new baseline acceptable heart rate of less than 100 beats per minute is generally recorded in the standing orders along with a directive against the use of beta blockers. The rate typically increases after each IL-2 dose, peaks at 2-4 hours and returns to baseline prior to the next dose. It is a compensatory response to hypotension, but is also a consequence of secondary cytokine and stress related epinephrine release and fever. It is important to distinguish the cause in order to avoid unnecessary fluid boluses. The tachycardia seems to become more pronounced and prolonged with an increased number of doses. It usually resolves over time between doses and is back to baseline before the next dose. Some centers extend the time between doses to allow recovery and others hold a dose until the next scheduled administration time.

Isolated premature ventricular contractions (PVCs) are not a contraindication to continued IL-2 dosing, but if the frequency increases (above 10/min), or patterns such as quadrigeminy or couplets or greater arise, holding IL-2 doses until recovery may be necessary. Less common cardiac arrhythmias requiring immediate intervention include supraventricular tachycardia with rapid response, atrial fibrillation with rapid response, and ventricular tachycardia. Patients may continue IL-2, if SVT or atrial fibrillation are converted/controlled, sinus rhythm is maintained or heart rate is controlled and there are no signs of cardiac damage. Ventricular tachycardia requires evaluation for cardiac damage and is an absolute contraindication to more IL-2. Rarely, evidence of myocarditis or pericarditis is noted clinically or on an EKG. This may reflect lymphocytic infiltration [25],[26]. If concerns exist regarding myocarditis or ischemia, then cardiac enzymes or troponin should be monitored at least daily and IL-2 discontinued if confirmed.

#### Dyspnea/Hypoxia

Management of dyspnea requires astute assessment and medical judgment as to causality. HD IL-2-related respiratory symptoms may develop in relation to local capillary leak or lymphocyte adherence to pulmonary vasculature or generalized fluid overload. However, patients with a smoking history, chronic obstructive lung disease or a history of asthma may have pulmonary symptoms related to bronchospasm, even without significant fluid overload. All of the preceding problems will be associated with decreased oxygen saturation. The treatment will vary according to the specifics. In addition, these patients may be febrile, uncomfortable and anxious and may be tachypneic on that basis without associated hypoxia. Finally, an important metabolic cause of non-hypoxic tachypnea is acidosis.

The frequency of these problems varies, and is related to the center's plan for management of hypotension - aggressive fluid resuscitation versus less fluid and early use of vasopressors. Diuretics are used by some sites, but not recommended by most. The adverse effects on blood pressure and kidney refractoriness within 4 hours of a recent IL-2 dose, make this a relatively futile and counterproductive measure. Hypoxia should be quickly treated with nasal cannula oxygen, with increased support as necessary to maintain oxygen saturation at 95%. In the context of hypotension with an increased demand on the myocardium, adequate oxygenation is critical and patients should not be maintained with suboptimal saturation waiting for recovery. Patients with obstructive pulmonary disease may benefit from regular nebulizer treatments with bronchodilators, and often they are able to continue IL-2. Elevation of the patient's bed is helpful. Performance of a chest radiograph should be reserved for patients with clinical indications for investigation.

#### Oliguria/Rising creatinine

**Figure 3:**
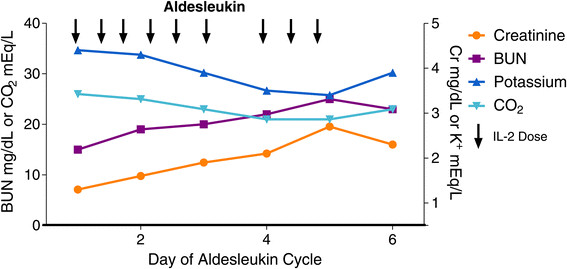
**Shows a week of HD IL-2 administration with typical evolution of daily serum BUN, creatinine, potassium and bicarbonate values.** In this patient the 1st and 2nd doses on day 3 are held. Spontaneous improvement occurs with conclusion of dosing on day 5.

Renal toxicity is believed to be related to a hypotension-induced pre-renal component as well as an intrinsic renal component mediated by secondary cytokines [29]-[31]. Most HD IL-2 physicians agree that renal toxicity is a universally reversible acute toxicity without chronic effects. It is manifest by oliguria and rising blood urea nitrogen and serum creatinine (Figure 3). All centers agree that renal toxicity reverses very quickly, even after withholding one dose of IL-2. All centers monitor renal function and urine output, but use quite different criteria for holding doses and different thresholds for concern. Criteria vary from minimum concern for a rising creatinine and a threshold of <100 ml/day urine output to creatinine thresholds of 2.5 and 10-15 ml of urine per hour, managed on a per shift schedule by fluid boluses. The typical range of serum creatinine considered for concern is from 3.0 to 5.0 mg/dL. Evaluation of the rate of rise may shed further light on the need for intervention.

There is enhanced concern for mRCC patients, due to the presence of only one kidney in many patients, and very rare cases from years ago in which patients suffered permanent renal damage. In patients with anuria and only one kidney, ureteral obstruction should be considered and ruled out. Dialysis is not indicated during treatment with HD IL-2. Discontinuing NSAIDS or nephrotoxic antibiotics when the creatinine is above 3.0 mg/dL is also prudent. The use of nephrotoxic contrast agents for imaging studies in patients receiving HD IL-2 should be not be prescribed as severe kidney damage may ensue.

### Examples

**Center A** - whose approach is to maintain blood pressure using relatively high volumes of fluid, both as maintenance and as boluses for hypotension, will hold doses of IL-2 for oliguria of less than 80-160 ml/8 hour, and give furosemide. Most centers do not utilize diuretics except on an as needed basis, since furosemide is only likely to be effective if given more than 4 hours after an IL-2 dose. If there is no diuresis, then IL-2 doses will be delayed.

**Center B** - who uses vasopressors and smaller volumes of fluids, will monitor 24 hour urine output and requires at least 100 ml/shift. If there is no improved output with holding a dose of IL-2 or if there is steeply rising creatinine, then another IL-2 dose will be held until the output is increased. Fluids will be given with vasopressors to attempt to improve output.

**Center C** - who uses a combination of fluids and vasopressors, also starts renal doses of dopamine from the beginning of treatment. However, a prospective randomized trial compared prophylactic use of low dose dopamine versus initiation of dopamine in response to oliguria, and found no benefit to prophylactic use [47]. Therefore, the efficacy of prophylactic renal dose dopamine is not confirmed. Dopamine may induce tachycardia which may prompt the holding of the next dose of IL-2.

#### Metabolic acidosis

Hypocarbia occurs for a multitude of reasons during HD IL-2 treatment: pulmonary injury, renal dysfunction, diarrhea, hypotension with high vasopressor levels resulting in poor tissue perfusion or dilution, due to the administration of non-bicarbonate containing fluids. Bicarbonate supplementation of IV fluids is recommended for serum bicarbonate levels of 19 mmol/L or an absolute drop of 4 mmol/L from baseline. If the serum bicarbonate remains < 18 mmol/L despite replacement, IL-2 should be held or stopped and bolus bicarbonate infusions administered. Low sodium bicarbonate has been associated with refractoriness to vasopressors which further decreases tissue perfusion, thus worsening the acidosis. It is important to replace the bicarbonate preemptively in order to avoid this circumstance. Some centers use lactated ringers solution for maintenance fluids and note their patients do not experience low serum bicarbonate levels. Most others utilize saline for fluid support and give replacement boluses of bicarbonate as needed. Whatever strategy is chosen it is critical not to fall behind in patients at greater risk.

#### Nausea/vomiting

Nausea/vomiting does not occur with all patients, but most centers prescribe scheduled prophylactic anti-emetics as well as "as needed" orders. However, some patients require intensive anti-nausea medication. The drugs preferred are ondansetron and granisetron, but some centers use prochlorperazine as initial treatment, only advancing to 5-HT3 antagonists as necessary. Patients who become symptomatic may benefit from conversion of oral medications to the intravenous route.

#### Mucositis

Mucositis does occur, but is not a problem for every patient. Therefore, it is managed on an as needed basis, with local medications such as non-alcohol containing mouthwash and toothpastes. Symptomatic mucositis may worsen upon discharge and may require additional management advice.

#### Diarrhea

Diarrhea can be minimal or extensive and varies from patient to patient. It is due to a combination of effects such as oral antibiotics, bowel edema from capillary leak syndrome, and other cytokine effects. It may be less of a problem than originally described perhaps due to continuous administration of 5HT antagonists. Diarrhea can complicate IL-2 administration due to fluid loss, electrolyte loss, local discomfort, inflammation, and even infection. Some centers postpone the night dose of IL-2 until the next morning, when diarrhea is severe to allow recovery until the next morning. Some centers convert oral antibiotics to IV, or switch to a single daily dose of vancomycin to reduce the possibility or impact of diarrhea.

#### Peripheral edema and weight gain

**Figure 4:**
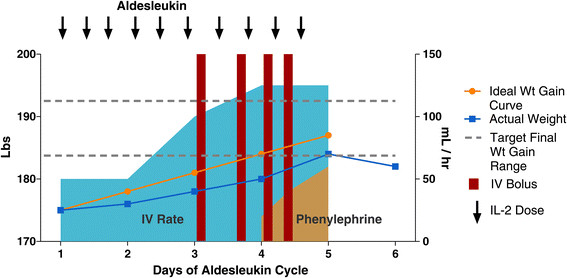
**Plots weight (actual and ideal curve), fluid support (IV rate and boluses) during a cycle of HD IL-2.** As the week progresses, weight gain falls behind ideal and increasing fluid and bolus support is necessary. Finally, vasopressor support is added before the final dose is administered.

Edema is a consequence of the capillary leak syndrome and fluid infusion, and in general causes discomfort but is not serious. Administration of fluids should result in a minimum 1-2 lb per day weight gain. Falling behind in fluid replacement may result in administering more fluid boluses, problems with oliguria and increased creatinine, and high doses of vasopressors ultimately leading to early cessation of IL-2 dosing. (Figure 4) Careful attention to daily weights and the rate of increase is important. After stopping IL-2, many centers choose to initiate diuresis prior to discharge, and may target achieving a weight that is within 5 pounds of admission. In general, diuretics are not effective until about 6-8 hours after the last dose of IL-2 and the cyclical effects of that dose begin to wane. If peripheral edema is present when the patient is otherwise ready for discharge, several centers send patients home with a prescription for diuretics, but others do not, advising the patient that diuresis will occur on its own. Mostly, this is a comfort issue, and patients who are very uncomfortable should receive a prescription for diuretics, usually 20 to 40 mg of furosemide depending on renal function, upon discharge with instructions to take until edema is gone or weight returns to the immediate pre-admission level.

#### Central nervous system neurotoxicity

All centers agree that this is a serious toxicity and one of the most difficult to anticipate. In particular, it is a toxicity that can worsen in the absence of additional IL-2 treatment, so it is important to identify early and withhold IL-2 therapy until its course can be established. Mild neurotoxicity, such as restlessness or insomnia, once treated with benzodiazepines, should not cause interruption of treatment with HD IL-2. Insomnia may be due to hospitalization and immobility or from IL-2, and appropriate medication should be considered. Severe neurotoxicity is a criterion for stopping IL-2 permanently. The progressive development of personality changes, hostility, confusion, disorientation, hallucinations, are criteria for stopping that cycle of IL-2 and may require intervention with anti-psychotic medications. Although generally occurring later in a cycle, gradual decay does not always occur, and an additional dose may cause a change from agitation to psychosis. Patients need to remain in the hospital until full recovery. Patients can be discharged on benzodiazepines for several days if their toxicity is limited to irritability. The most important management of CNS toxicity is continued reassurance and occasionally benzodiazepines or haloperidol can be considered.

#### Peripheral neuropathy

Peripheral neuropathy is uncommon but can be quite debilitating particularly in the upper extremities and may limit IL-2 dosing. This is due to an entrapment/compression syndrome in the wrist or forearm, associated with edema [48]. Pain medications (usually codeine or oxycodone) or medications more specific for neuropathy (gabapentin) may be helpful.

#### Cutaneous toxicity

Macular rash, redness and dry skin are common during IL-2 treatment, and progress as the week of treatment continues. All centers provide topical medications during treatment such as emollients, non-alcohol containing lotions, or non-steroid containing lotions or creams. Skin toxicity is particularly noticeable in patients with fair skin, and generalized exfoliation and peeling of hands and feet can occur, often after patients are discharged from the hospital. Lotions and emollients should be started at or before the first sign of erythema.

Pruritus is not directly related to the degree of exfoliation. A small study has shown that pruritus is not related to the eosinophilia that sometimes develops with HD IL-2 administration. Pruritus may be cytokine mediated, as has been shown in cutaneous T-cell leukemia in which T-cells infiltrate the skin, or due to hyperbilirubinemia (49). Although antihistamines are used for IL-2 induced pruritus, one study has reported, and anecdotal reports have suggested, that gabapentin has a major beneficial effect on pruritus [49]. Itch nerve fibers are pain fibers and gabapentin is effective in peripheral neuropathy. If the pruritus is cytokine mediated, and reflects irritation of peripheral nerves, then gabapentin would be expected to be beneficial. In severe cases opioids may be beneficial as the pain fiber-mediated itch may respond to central acting agents. In patients where the opiates administered for rigors are suspected as the cause, naltrexone may be used to treat the itching. Pruritus is not dose-dependent. It may not be resolved in one week if the patient returns for the second cycle of IL-2 treatment, and may be criteria for a delay. Skin management is an important part of discharge planning.

#### Electrolyte abnormalities

A variety of electrolyte abnormalities are noted, including hypomagnesemia, hypocalcemia, hypokalemia, hypophosphatemia and hyponatremia. These will progressively worsen during the treatment and require careful correction (Figure 3). Most centers correct the magnesium and potassium, and monitor the others. Often the serum calcium is normal after correcting for hypoalbuminemia.

#### Hematologic toxicity

**Figure 5:**
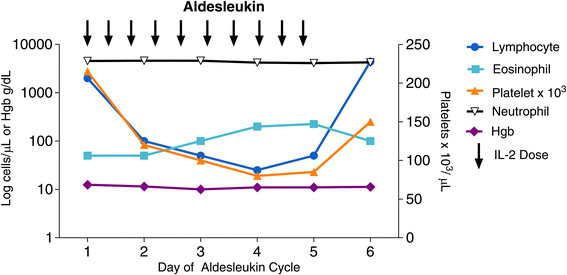
**A logarithmic plot of blood lymphocyte, eosinophil, platelet and granulocyte absolute counts and hemoglobin during a cycle of HD IL-2 administration.** Platelet and lymphocyte counts fall rapidly to very low levels. Eosinophils progressively rise during administration. After cessation of treatment, platelets and lymphocytes quickly return to normal and often rebound to 2-5 times their baseline levels. Eosinophilia may persist for weeks.

Acute changes in the cellular elements of the blood are expected and may be dramatic (Figure 5). Thrombocytopenia is a common toxicity of HD IL-2 therapy. However, it rapidly reverses once IL-2 is discontinued and does not require intervention in most cases. Patients with mM who have received prior chemotherapy are at risk for leukopenia during IL-2 therapy. It is not yet clear if mRCC patients who have previously received anti-VEGF TKIs are at risk of leukopenia.

One group studied the bone marrows of IL-2 patients who developed thrombocytopenia and found normal numbers and morphology of megakaryocytes. They attributed the thrombocytopenia to sequestration in the spleen due to IL-2-induced splenomegaly (JPD, personal observation). Lymphocytes similarly disappear within hours after the first dose, probably due to margination and diapedesis into the tissues. Coagulation abnormalities have also been reported. Fibrinogen and d-dimers were normal, thus arguing against consumption or microthrombi and suggesting impaired hepatic synthesis [50]. Coagulation abnormalities and other hematologic toxicities rapidly reverse with discontinuation of IL-2 and do not require altered dosing of IL-2. Anemia can occur but rarely are transfusions required. In fact, if patients become significantly anemic during IL-2 administration, other causes, such as hemorrhage, should be sought.

#### Hepatic enzyme and function abnormalities

The most frequent abnormalities noted are low albumin (both from capillary leak and decreased synthesis), mild coagulopathy, hyperbilirubinemia, and less commonly hepatic enzyme elevations [51],[52]. The bilirubin may rise to approach 10 mg/dL and the albumin drop to 2 gr/dL or less. These will reverse once IL-2 is discontinued. Some sites discontinue acetaminophen if the bilirubin becomes high, most do not. There does not appear to be a threshold for bilirubin abnormalities for holding IL-2 or delaying IL-2. Elevation of hepatic enzymes does cause some concern when the value is 5-10 fold above normal and some centers hold IL-2 for increases of this magnitude.

#### Criteria for holding or stopping HD IL-2

The main criteria used for holding and/or stopping IL-2 treatment are summarized in Table 3. The severity of adverse events associated with bolus IL-2 peaks 4-6 hours after a dose and most interventions will be employed at that time. It is critical to re-evaluate the patient before receiving each dose to make sure that many of these events are improving or have subsided at that time. Holding a dose of IL-2 is the preferred management of prolonged toxicity from a previous dose. There are no individual dose reductions. If a toxicity occurs early in the cycle and returns to near baseline, such as resolution of elevated heart rate or low blood pressure, then treatment can resume. However, toxicity is cumulative over 5 days, both in extent and duration. In addition, toxicities become increasingly refractory to treatment. Thus, later in the week, more intensive or sustained toxicities become reasons to stop that cycle of IL-2. Recommendations for delaying, withholding or stopping IL-2 doses have evolved from experience and are outlined below and in Table 3.

**Table 3:** **Criteria for holding and stopping IL2** ^**#**^

System	Relative criteria	Absolute criteria
Cardiovascular	Sinus tachycardia 100-130 BPM*	Sinus tachycardia > 130 BPM*
		ECG indications of ischemia
		Atrial fibrillation
		Supraventricular tachycardia
		Ventricular arrhythmias**
		Elevated CKB-MB isoenzyme of troponin levels
Dermatologic	Moist desquamation	
Gastrointestinal	Diarrhea, 1000 ml/shift	Diarrhea 1000 ml/shift x 2
		Vomiting not responsive to medication
	Ileus/abdominal distention	Severe abdominal distention affecting breathing
	Bilirubin > 7 mg/dL	Severe, unrelenting abdominal pain
Hemodynamic^#^*	Maximum Phenylephrine 1-1.5 mcg/kg/min	Maximum Phenylephrine 1.5-2.0 mcg/kg/min
	Minimum Phenylephrine > 0.5 mcg/kg/min	Minimum Phenylephrine > 0.8 mcg/kg/min
Hemorrhagic	Sputum, emesis, or stool heme-positive	Frank blood in sputum, emesis, or stool
	Platelets 30,000-50,000/mm^3^	Platelets < 30,000 mm^3^
Infectious		Strong clinical suspicion or documented
Musculoskeletal	Weight gain > 15%	
	Extreme tightness	Extreme paresthesias
Neurologic	Vivid dreams	Hallucinations
	Mild anxiety	Persistent crying
		Mental status changes not reversible in 2 hours
		Unable to subtract serial "7 s" or spell "WORLD" backward
		Disorientation
Pulmonary	Resting shortness of breath	> 4 L O_2_ by nasal cannula or 40% by mask to maintain ≥ 95% O_2_ saturation
	3**-** 4 L O_2_ by nasal cannula for O_2_ saturation ≥ 95%	Intubation
	Rales 1/3 up chest	Moist rales ½ up chest
Renal	Urine output 80-160 ml/shift	Urine < 80 ml/shift
	Creatinine 2.5-2.9 mg/dL	Creatinine ≥ 3 mg/dL
		HCO_3_ ≤ 18 meq/dL

Abbreviations: BPM, beats per minute; ECG, electrocardiogram.

*Persistent at the time of dosing after correcting hypotension, fever, and tachycardia and discontinuing dopamine.

**Including premature ventricular contractions, bigeminy, and tachycardia.

^#^In order to maintain acceptable BP and pulse criteria.

*Phenylephrine Max is during the interval, Min at the time of dosing.

#The above criteria should be assessed at the scheduled time of next dose after aggressive measures to correct toxicity have been undertaken.

If ≤ 3 relative criteria, delay dosing, continue corrective measures and administer another dose if recovered in < 24 hours.

If >3 relative criteria or an absolute criteria are observed stop IL-2 for the current cycle.

### Cardiac

#### Holding

Sinus tachycardia is the norm after IL-2, but this should return to a pre-defined baseline before administering further doses of IL-2. If the heart rate remains above 100-120 beats per minute after correcting hypotension in an afebrile patient, when a dose is due, that dose should be delayed or skipped.

#### Stopping

Sustained rapid (>130-140/min) supraventricular tachycardia or rapid atrial fibrillation will require intervention, and stopping of IL-2, at least for the current cycle. If the supraventricular arrhythmia can be converted to NSR quickly or the heart rate and blood pressure managed despite atrial fibrillation, then consideration could be given to resuming IL-2 treatment. Once treated, IL-2 may be administered in a subsequent cycle. Ventricular tachycardia or evidence of ischemic myocardial damage are indications for permanently stopping IL-2 treatment. A diagnosis of myocarditis should prompt discontinuation of that cycle of IL-2, but if LVEF returns to baseline, patients may cautiously proceed with additional cycles of HD IL-2, usually without recurrence of this side effect.

### Hypotension

#### Holding

IL-2 dosing should be delayed until hypotension recovers from the previous dose and the vasopressor level (if applicable) is at a baseline established for dosing. If hypotension requires additional fluid boluses then an assessment of respiratory function will help determine when the next dose can be administered. If high doses of phenylephrine continue to be required, some patients will respond to replacing phenylephrine with norepinephrine at low doses (prefer 2-4 mcg/kg/min but may titrate up to 10 mcg/kg/min for short periods of time). If high-doses of vasopressors are needed, IL-2 should be discontinued.

#### Stopping

The degree and duration of significant hypotension progresses during the week, and thus may require more fluid or higher doses of vasopressors to maintain adequate blood pressure. When hypotension requires maximum doses of vasopressors or a change to alternate vasopressors, such norepinephrine, IL-2 should be stopped. IL-2 should also be stopped if fluid overload precludes using fluids for maintaining blood pressure, as pulmonary reserve may be compromised.

### Neurologic

#### Holding

Personality changes can be subtle, and it may be difficult to distinguish when they are the result of IL-2 or from other causes. However, if staff or family observe a change in personality, exhibition of agitation or emotional lability, IL-2 neurotoxicity must be considered. Improvement of mild neuropsychiatric complications after drug therapy or after delay of a dose of IL-2 is a sign to then continue dosing.

#### Stopping

As a precipitous decline may occur with additional IL-2 doses, lack of improvement with specific therapy and passage of time warrants stopping therapy. The progression of personality changes or agitation to extreme emotional lability, crying, aggressiveness, disorientation, or hallucinations are absolute criteria for stopping IL-2. Such symptoms of neurotoxicity may require medication and continued hospitalization until resolution. Rarely peripheral compression neuropathy or vasospasm occurs, resulting in finger pain or paresthesias poorly responsive to medication which may also require stopping IL-2. This is more common during second or subsequent courses of therapy.

### Pulmonary

#### Holding

Dyspnea and hypoxia reflect capillary leak and can lead to a requirement for oxygen to maintain an oxygen saturation of 95%. Oxygenation should be carefully monitored and maintained. Patients may develop tachypnea and rales. Tachypnea may also result from acidosis or fever which should also be corrected. Delaying IL-2 administration will allow diuresis spontaneously or with diuretics, and after improvement, it may be possible to administer additional IL-2. Patients may develop wheezing despite nebulizers and this may reflect capillary leak or bronchospasm or both. Careful diagnosis and aggressive correction of pulmonary symptoms should occur quickly. The severity will influence the decision to delay or discontinue IL-2 therapy.

#### Stopping

Patients with dyspnea or hypoxia requiring more than 4 liters of oxygen to maintain saturation of 95% or who are clearly fluid overloaded are at risk for continuing IL-2. Intubation should not be necessary with current guidelines, but is an absolute indication for stopping IL-2.

### Oliguria/creatinine

#### Holding

Pre-established criteria at each center are used to determine if urine output and serum creatinine are adequate to administer another dose. Some centers require urine output of 30 ml/hour, others 80-160 ml/8 hour shift. Some centers delay a dose of IL-2 when urine output has fallen, and resume IL-2 when urine output is evident. Some centers will only delay a dose if oliguria is accompanied by other signs of significant toxicity, such as fluid overload that does not allow further fluid replacement. Elevated serum creatinine concentration alone is usually not a reason for delaying a dose of IL-2, since it is primarily a reflection of hypotension, capillary leak, and/or oliguria. However, many centers set a threshold level for serum creatinine, and will stop IL-2 treatment if the level rises above 3 to 5 mg/dL. Dialysis is not necessary, as renal function returns upon cessation of IL-2 administration.

#### Stopping

Each center will set its criteria for tolerability of oliguria and/or serum creatinine level with which to hold or stop IL-2. This is related to the degree of capillary leak, the rate of rise of serum creatinine, the degree of oliguria, and responsiveness to holding a dose of IL-2.

### Diarrhea

#### Holding

If diarrhea becomes very frequent, such as 5-6 movements/day of any volume, most centers would delay IL-2 therapy to allow some recovery. If diarrhea improves with delaying doses, IL-2 may continue, but if as a consequence of the diarrhea, the patient develops local pain and irritation and fluid loss and electrolyte imbalance are apparent holding another dose of IL-2 is prudent. If there is concern about infection, treatment may have to be discontinued. Electrolytes should be replaced as needed, particularly bicarbonate, prior to resuming IL-2. Patients who have had prior treatment with ipilimumab and develop diarrhea while on high dose IL-2 should be evaluated for inflammatory colitis.

#### Stopping

Rarely, continued large volume or frequent diarrhea despite medical intervention requires stopping IL-2. Electrolyte loss may not be compensated by replacement and due to the depressed renal function overshoots of potassium are to be avoided. If there is voluminous diarrhea, examination for hemorrhagic diarrhea or *C. difficile* toxin should be obtained. This is rare, but must be ruled out in this setting.

### Metabolic acidosis

#### Holding

This can be a result of diarrheal losses of bicarbonate or lactic acidosis due to tissue hypoperfusion. If replacement controls the level of serum HCO3, then IL-2 can be continued. Utilization of lactated Ringer's instead of half normal saline for maintainance fluid may prevent low serum bicarbonate levels. The occurrence of a serum HCO3 level of < 18 meq/dL is a worrisome sign and must be promptly addressed. If it does not quickly respond to bicarbonate replacement then an alternative source of acid production, such as bowel ischemia, should be considered and sought. For centers that use Ringers Lactate as maintenance intravenous fluids, the need for bicarbonate boluses has largely been eliminated, as significant acidosis has not been observed.

#### Stopping

Serum bicarbonate of < 18 meq/dL unresponsive to replacement therapy is reason for stopping IL-2.

### Hematologic

#### Holding

Changes in blood counts are usually mild and not a reason to hold IL-2. Platelet counts have been seen as low as in the 20,000/mm^3^ range, but rapidly recover after stopping IL-2. Some centers will transfuse, however, and stop on the basis of thrombocytopenia, if more than 10 doses have been administered.

#### Stopping

There is almost never a reduction in hemoglobin sufficient to transfuse, so if that occurs, a search for additional causes should be made. Examples may include bleeding and in one case, pure red cell aplasia was noted in the marrow immediately following IL-2 therapy (JPD, personal communication).

### Infection

#### Stopping

Any documented gastrointestinal or central line infection or suspected sepsis is a criterion to stop IL2. Central venous catheters should be promptly removed as line infection can quickly proceed to septicemia particularly if *Staphylococcus aureus* is the cause.

### Cutaneous

#### Delay cycle

If severe cutaneous toxicity does not resolve mid-cycle, a delay in the second week is sometimes necessary.

#### Stopping

Rarely pruritus or cutaneous toxicity can accelerate, particularly during the second cycle of IL-2 treatment, and the sensation may become intolerable, despite treatment. This may be a criterion for stopping due to patient discomfort.

### Malaise/intolerance

#### Stopping

Patients may state that they cannot tolerate more treatment and most IL-2 oncologists will stop IL-2 in the face of extreme agitation or when patients do not wish to continue.

### Other

Clearly if there are issues that require transfer of a patient to an ICU because of decompensation then IL-2 should be discontinued at that time. This recommendation does not apply to patients electively treated in an ICU setting from the very beginning or when routine transfer to an ICU is necessary for the use of vasopressors.

### Discharge management

Discharge planning should continue throughout the hospitalization, to familiarize the patient and home caregivers with routines and expectations during the first few days home. Once IL-2 is discontinued, patients recover very quickly from its effects despite the cumulative toxicity experienced during the cycle. Usually by 12 hours after the last dose of IL-2 patients are anxious to leave the hospital and return home. If they are still requiring vasopressors, they must be weaned off and blood pressure must be stable while they ambulate with assistance. They should be prepared to sleep for several hours upon returning home since they will still be experiencing significant fatigue. Most sites advise patients not to drive or use dangerous equipment for several days. Vivid dreams may occur during the first several nights home which may be frightening. Sleep medication may be needed short-term.

The serum creatinine usually levels off or begins to fall by the time of discharge, but if it is still elevated, it usually will normalize within the next several days. Some centers prefer to recheck BUN and creatinine after discharge, but experience has shown that this is unnecessary.

At discharge, patients will still have fluid overload, mostly peripheral, and some centers provide a prescription for diuretics to be used as needed, particularly if diuretics were necessary in the hospital after IL-2 cessation. Rarely, patients have fluid shifts from the periphery into the pulmonary capillary bed, and develop late dyspnea requiring diuresis. Patients receiving diuretics should be instructed not to take them until they arrive at home, in order to minimize risk of emergency stops on the way home from the hospital.

Patients who have pre-existing hypertension will need to resume their medications during the week between cycles, usually starting 48 hours after discharge, and they will have to stop these medications within 48 hours prior to their next admission for IL-2. If patients are planning further IL-2 treatment they should not re-start beta-blockers.

For those patients who experience cutaneous manifestations, the erythematous rash may evolve into extensive peeling of the epidermis after hospital discharge, particularly involving the palms and soles. Pruritus may worsen after discharge, and creams should be used liberally as well as increasing doses of gabapentin and potentially opioids. Uncommonly, skin excoriation as a result of pruritus may delay the second cycle of IL-2, if there are open lesions and severe redness.

Hypothyroidism, sometimes preceded by an episode of hyperthyroidism, can occur weeks or months after IL-2 therapy and thyroid function should be monitored during regular follow-up visits [53]-[55]. If a patient returns for IL-2 therapy and is severely hypothyroid, hormone replacement should be initiated. IL-2 should be delayed until a euthyroid state is achieved on a stable replacement dose.

Patients who are used to aerobic physical activity will note that their stamina and lung capacity are less, immediately following IL-2. This will improve as they recondition. These are delayed effects of capillary leak and cytokines on the cardiopulmonary systems that spontaneously resolve.

The patient should be warned that iodinated contrast media used in radiographic studies have caused skin and systemic reactions in patients who have received prior HD IL-2 [56]. These reactions may be intense and mimic the side effects of IL-2, including oliguria and elevation in creatinine levels. Hence, using iodinated contrast agents immediately prior to retreatment with IL-2 should be avoided.

Follow-up phone contact is important in the first one to two weeks following IL-2 treatment, followed by regular follow-up visits with scans to assess disease status. Follow-up scans are usually obtained 6-12 weeks after a full course of IL-2 therapy is completed to assure that there is no rapid growth of disease. Sometimes this scan will in fact show an early response, or small growth with subsequent response at the next scan. A second scan at 12-16 weeks will confirm whether benefit has occurred. However, there have been patients who have demonstrated continued response scan by scan over the course of a year, and others who were stable for 6 months, and then showed tumor regression. Other anecdotal reports describe patients who have been stable and had residual, previously biopsied disease resected, which was found to be only scar tissue. Although the natural course of response after IL-2 is not well defined, it may be important to allow sufficient time for immunotherapy to work before changing treatment approaches such as IL-2, as other forms of immunotherapy has been associated with delayed kinetics of tumor regression. Additionally for patients who remain stable after IL-2 therapy, this appears to be of clinical benefit and such patients may be monitored or retreated [9],[10],[13].

Relapse of disease in patients that have previously responded to IL-2 generally raises the question of retreatment with IL-2. However, multiple factors will need to be considered, such as the amount of prior IL-2 received, the intensity of side effects experienced, and other treatment options. In addition, it should be noted that retreatment with IL-2 alone in this setting may be less effective [57].

### Establishing HD IL2/immunotherapy centers

#### Clinical requirements

All personnel need to have specialized training in managing HD IL-2 patients. Usual maneuvers to treat patients with cytokine-induced systemic responses, such as sepsis, are managed very differently than HD IL-2 patients. Anticipation of the expected side effects, and rapid appropriate correction are central to management of these patients. Treating a minimum number of patients per year is important, as quality depends upon familiarity and repetition.

##### Nursing requirements

The optimal scenario is that there is sufficient nursing staff to have a reduced patient ratio on all shifts such as 1 nurse to 2-3 patients. The lower nurse to patient ratio of 1-2 may be necessary during day 3, 4, or 5 of IL-2 treatment when the patient is experiencing more varied and intense IL-2 side effects. This may be less critical as the nursing staff becomes more familiar with the management of HD IL-2 patients. It is preferable to have cardiac monitoring, with BP, HR, and oxygen saturation panels available for regular use. Intensive in-service training should be provided for all shifts and for new staff as needed. Patient comfort and education are important nursing goals [58].

##### Physician commitment

It is preferable that more than one physician be trained and accept responsibility for patient management. In some centers a group of only medical oncologists or oncologic surgeons are responsible and in others centers an oncologist will share management with cardiology and pulmonary staff, either on the oncology unit or in the ICU. Others utilize specially trained hospitalists or even oncology hospitalists for IL-2 management, particularly at night. Nurse practitioners or physician assistants have also maintained coverage in some centers with backup support from a covering medical oncologist.

##### Immunotherapy coordinator

This person(s) is usually a nurse who is responsible for organizing the pre-screening of patients for IL-2 eligibility, as well as obtaining insurance approval. In most centers, this individual is also involved in discharge management and follow-up for IL-2 patients and is fully aware of the special needs of their management and follow-up.

### Administrative issues

Prior to establishing the HD IL-2 treatment program, there must be an evaluation of institutional policies regarding cardiac monitoring and use of vasopressors on a regular oncology unit, versus a requirement for ICU care. Management of HD IL-2 is much more akin to a surgical procedure where the patient is admitted with an expectation of a standard length of admission and utilization of a predictable amount of hospital resource with a discharge home of a convalescing patient. If IL-2 is to be given on a regular unit, an assessment of what type of nursing training will be required for cardiac monitoring, frequent vital signs, and vasopressor management will need to be made. Attention to integrating clinical assessment and pharmacy preparation of the drug is also important to avoid wasting drug or inadvertent administration to a patient experiencing a significant toxicity. Based on these issues, the following two key questions will need to be addressed: Where in the hospital will HD IL2 be administered? Who will be responsible for patient management?

Regardless of the setting, low nurse/patient ratios, experience in managing complex acutely ill patients and consistent practice in these settings is critical. HD IL-2 specific training for all personnel is essential to success. Hospital policies and availability of resources often determines where HD IL-2 is administered.

#### ICU setting

Some centers only use vasopressors in an ICU. Nursing staff levels may also be an issue for non-ICU management in some institutions. ICU nurses must be specifically trained in management of IL-2 patients, as HD IL-2 management decisions are often counter intuitive to standard ICU practice. Staff should comply with chemotherapy certification if hospital policy dictates, but it is not necessary. In the ICU, there must be clear delineation of which physician team will have primary responsibility for the patients. Ideally, it should be the HD IL-2 team, rather than the ICU physicians.

Hematopoietic Stem Cell Transplant/HD IL-2 Unit: A high intensity oncology unit is available at some centers. IL-2 treatment and stem cell transplant both require intensive nursing, monitoring and management. This is a natural setting for HD IL-2.

#### Inpatient oncology unit

Experienced high volume centers often administer all HD IL-2 on a standard oncology unit equipped for cardiac monitoring. Alternatively, sites may start treatment on an oncology unit and transfer to ICU when it is necessary to monitor and administer vasopressors. If transfer to an ICU does occur, the same medical team should have primary responsibility for the care of the patient.

All these facets of care should be clearly defined and agreed upon by all parties before training, instruction, and treatment begins.

### Financial/Billing issues

There must be an education of medical records and billing staff to allow optimal documentation for coding and billing for the level of intensity of HD IL-2 administration. They must be cognizant of the dedicated DRG and CPT codes for IL-2 administration. A training session can be provided for instruction in recognizing the specific terms and converting to specific coding for IL-2.

Medical and nursing personnel who are documenting care and management of these patients need to be trained in carefully documenting the clinical issues and the severity of illness during IL-2 administration.

Some institutions will require a cost analysis prior to initiating and supporting an IL-2 program.

## Conclusions

High dose interleukin-2 remains an important component of the therapeutic armamentarium for patients with metastatic renal cell cancer and metastatic melanoma. A subset of these patients continue to achieve a consistent durable complete response that can last for decades. The authors agree that peri-treatment mortality can be reduced to less than 1% when guidelines are diligently followed. High priority research includes an exploration of combination therapy wherein IL-2 is used with other immunotherapy agents, stereotactic radiation, or new targeted therapeutics being developed for these diseases [44],[59]. Another high priority is the identification and validation of predictive biomarkers to better select potential responders prior to treatment. The education of more physicians in how to administer HD IL-2 and manage patients on treatment can increase the number of patients who might benefit from the therapy. This Best Practices manuscript has been developed to further enhance guidance for administration of HD IL-2 by surveying the experience of more than 20 experts with years of experience in its utilization.

## Authors' contributions

All authors contributed to the manuscript writing and gave final approval to the manuscript. HLK, DJS, AAT, SSA, MBA and JNL participated in a 2 day roundtable discussion of managing patients receiving HD IL-2 with the intention of creating best practices. JPD and JNL assembled the information from the conference into a draft manuscript that was reviewed and revised by all authors.

## Additional file

## Electronic supplementary material

Additional file 1: Standing order example. (DOC 34 KB)

## Authors’ original submitted files for images

Below are the links to the authors’ original submitted files for images. Authors’ original file for figure 1
Authors’ original file for figure 2
Authors’ original file for figure 3
Authors’ original file for figure 4
Authors’ original file for figure 5

## Abbreviations

HD: High dose
CR: Complete response
IL-2: Interleukin-2
mRCC: Metastatic Renal Cell Carcinoma
mM: Metastatic melanoma
LAK: Lymphokine activated killer
LVEF: Left ventricular ejection fraction
TNF-α: Tumor Necrosis Factor Alpha
BUN: Blood urea nitrogen
NCI: National Cancer Institute
IU: International units
IU/kg: International units/kilogram
CWG: Cytokine Working Group
ICU: Intensive Care Unit
ECOG: Eastern Cooperative Oncology Group
K: Karnofsky
PS: Performance status
CAD: Coronary artery disease
VEGF: Vascular endothelial growth factor
TKI: Tyrosine kinase inhibitor
EKG: Electrocardiogram
PRN: As needed
NSAIDS: Non-steroidal anti-inflammatory drugs
mg/dL: Milligram/deciliter
SIRS: Systemic inflammatory response syndrome
mmHg: Millimeter of mercury
BP: Blood pressure
mcg/kg/min: Microgram/kilogram/minute
LR: Lactated ringers
HR: Heart rate
PVCs: Premature ventricular contractions
SVT: Supraventricular tachycardia
mmol/L: Millimole/liter
5-HT3: 5-hydroxytryptamine
lb: Pound
mg: Milligram
CNS: Central nervous system
g/dL: Gram/deciliter
LVEF: Left ventricular ejection fraction
HCO3: Bicarbonate
mm: Millimeter
DRG: Diagnosis related group
CPT: Current procedural terminology
VEGFR: Vascular endothelial growth factor receptor
