# Supplementary material for: High dose interleukin-2 (Aldesleukin) - expert consensus on best management practices-2014
Source: J Immunother Cancer. 2014 Sep 16;2:26. doi: 10.1186/s40425-014-0026-0 (PMC6889624; doi:10.1186/s40425-014-0026-0)
Supplement: Supplementary file 1 — Additional file 1: Standing order example. (DOC 34 KB) [file 40425_2014_26_MOESM1_ESM.doc]

Additional file 1. Standing Order Example

## High Dose Interleukin-2 Admission Orders Page 1 of 2

1. **Admit to 7N**—Dr.
2. **Diagnosis**: Renal Cell Cancer / Melanoma Wt: __________ kg
3. **Allergies**:
4. **Vital Signs**: On admission and q2h while on IL-2, incl. O_2_ sat. Daily weight at 0600
5. **Activity**: OOB with assistance only. Bed alarm enabled.
6. **Nursing**:
   1. Strict I/O
   2. Start oral care program with NS rinses qid
   3. Geomatt prn immobility
   4. Notify MD/NP for: SITS > 2, T >104.0 ºF, RR > 30/min, SaO_2_ <90% on >4 L O_2_. Apical pulse >140 (not r/t fever) or new onset of irregular pulse—obtain 12-lead ECG and call MD/NP with results (call if ECG done within 24 hours). **Critical abnormal lab values**: including CO_2_ <20 Magnesium <1.4, Phos <1.5, K^+^ <3 or >5.5, Ca^++^ <7.0, platelets <20,000, Hgb <8.0.
7. **Diet**: Regular
8. **IV**: D_5_W @ 10cc/hr. May hold between IL-2 doses.
9. **O_2_** prn to keep SaO_2_ ≥95%. Notify MD/NP prior to next IL-2 dose if patient on O_2_.
10. **Catheter:** A temporary triple lumen catheter for high-dose IL-2 administration will be placed prior to first IL-2 dose by Interventional Radiology.
    1. **Lorazepam 1 mg PO x 1 prn** prior to procedure
11. **Labs:**
    1. **Admission labs: CBC, CMP, Phosphorus, Magnesium, LDH, Serum bHCG** on admission (for all pre-menopausal females)
    2. **Daily Labs: CBC, CMP, Phosphorus, Magnesium, CPK**

# TREATMENT MEDICATIONS

1. **IL-2 (Aldesleukin) 600,000 International Units/kg X _____kg =_________ million international units** to be administered by IV infusion over 15 minutes q8h starting on __________ for five days (max total doses = 14). Reconstitute IL-2 with sterile water and dilute with either 15 or 20 ml of D_5_W in 50 ml syringe.
2. **Acetaminophen 650 mg PO tablets or elixir q4h** starting before and during IL-2 treatment (PR may be substituted for PO if patient unable to take PO).
3. **Famotidine 20 mg PO/IV q12h** during IL-2. May substitute **Omeprazole 20 mg PO daily** if patient on a proton pump inhibitor prior to admission.
4. **Ibuprofen 600 mg PO tablet or suspension q6h** during IL-2.

D/C Ibuprofen if creatinine is >3.0. Change famotidine to 20 mg q24h if creatinine >2.0. D/C acetaminophen/famotidine/ibuprofen 12 hours after last IL-2 dose.

1. **Cephalexin 250 mg PO q8h** if central venous catheter in place **(cefazolin 1 gram IV q12h** may be substituted if unable to take PO).

### ORDERS CONTINUED

Physician’s Signature: _______________________________ Date/Time: ______________

***ALL VERBAL ORDERS MUST BE SIGNED/DATED/TIMED BY AUTHORIZED PRACTITIONER,**

### NO LATER THAN THE DATE THE RECORD IS CLOSED.*

## High Dose Interleukin-2 Admission Orders Page 2 of 2

## PRN MEDICATIONS

1. **STEROIDS WILL NOT BE PERMITTED.**
2. **Electrolyte replacement** for **AM** labs: Call first if Creatinine >6:

a. For Magnesium <1.6 give **Magnesium Sulfate 2 gm IV x 1**

1. For Calcium <8 give **Calcium Gluconate 1 gm IV x 1**
2. For Phosphorus <2 give **Sodium Phosphorus 20 mmo IV over 6 hours x 1**
3. **Meperidine 25 mg slow IV push q 5 minutes** prn rigors—NTE 200mg/2hr.
4. **Hydromorphone 1-2 mg PO/IV q3h** prn chills or prn pain.
5. **Oxycodone 5-10 mg PO q3h** prn pain**.**
6. **Prochlorperazine 5-10 mg PO/IV q6h** prn nausea/vomiting
7. **Ondansetron 8 mg PO/IV q8h** prn nausea
8. **Metoclopramide 10 mg PO/IV ac and hs** prn nausea, symptoms of gastric stasis—d/c if diarrhea develops.
9. **Alteplase** per standard dosing procedure.
10. **Zolpidem 5 mg PO hs** prn insomnia. MR x 1 one hour after 1^st^ dose.
11. **Diphenhydramine 25-50 mg PO/IV** or **hydroxyzine 25 mg PO q4h** prn pruritus.
12. **Loperamide 2 caps PO q4h** until diarrhea stops for 8 hrs (NTE 12/day).
13. **Miracle Mouthwash** prn mouth soreness
14. **Eucerin Cream** or **ammonium lactate lotion** to skin prn pruritus and dry skin.

## Bolus Orders

1. **If SBP drops below _____mm Hg or < 90 and symptomatic**: Administer 250 cc bolus of NS over 15 minutes. May repeat x 2 prn within 24 hours. If SBP still <____ after 3^rd^ bolus see below:

## Monitor Orders

1. **If SBP is <_____ mm Hg after 3^rd^ bolus** / 24 hrs start phenylephrine 40 mcg/min IV (phenylephrine 40 mg in NS 250 ml) and begin continuous cardiac monitoring. Notify MD/NP.
   1. Titrate to maintain SBP >85-90. Increase dose by 25-50 mcg q 5-15 minutes until >90 systolic (see procedure—High-dose IL-2 continuous cardiac monitoring).
   2. Use minimum phenylephrine dose to maintain target BP.
   3. Pharmacy may concentrate infusion solution as needed.
2. **Notify MD/NP** if phenylephrine dose >200 mcg/min is required to maintain SBP > 85-90
3. **Phenylephrine dose of 4 mcg/kg = _____________________________________**
4. **Notify MD/NP** of patient status prior to each dose of IL-2. ****An MD/NP order** must be obtained for each IL-2 dose if patient is on phenylephrine and/or cardiac monitoring.
5. **Place monitor strip** on chart initially and at the beginning of each shift.
6. **Activity**: No out of bed activity except to bedside commode or chair/recliner with assistance. No showers, sponge bath only.
7. **After last dose of IL**-2, titrate off phenylephrine and discontinue cardiac monitoring if vital signs stable 2 hours after phenylephrine infusion discontinued.

Physician’s Signature: _______________________________ Date/Time: ______________

***ALL VERBAL ORDERS MUST BE SIGNED/DATED/TIMED BY AUTHORIZED PRACTITIONER,**

### NO LATER THAN THE DATE THE RECORD IS CLOSED.*
