# Supplementary figures and images for: High dose interleukin-2 (Aldesleukin) - expert consensus on best management practices-2014
Source: J Immunother Cancer. 2014 Sep 16;2:26. doi: 10.1186/s40425-014-0026-0 (PMC6889624; doi:10.1186/s40425-014-0026-0)

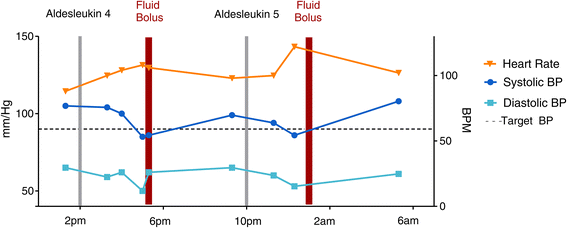

Supplement: Supplementary file 2 — Authors’ original file for figure 1 [file 40425_2014_26_MOESM2_ESM.gif]

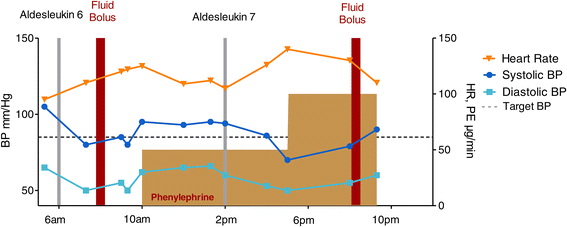

Supplement: Supplementary file 3 — Authors’ original file for figure 2 [file 40425_2014_26_MOESM3_ESM.gif]

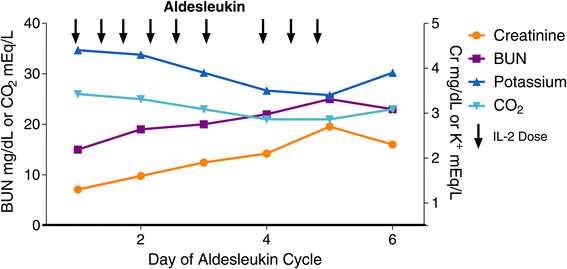

Supplement: Supplementary file 4 — Authors’ original file for figure 3 [file 40425_2014_26_MOESM4_ESM.gif]

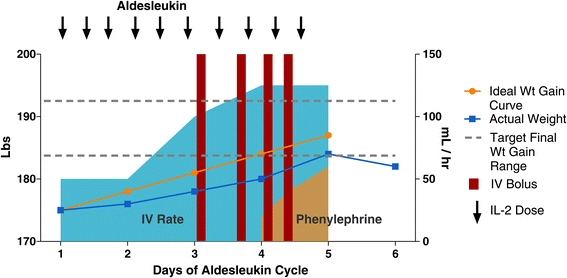

Supplement: Supplementary file 5 — Authors’ original file for figure 4 [file 40425_2014_26_MOESM5_ESM.gif]

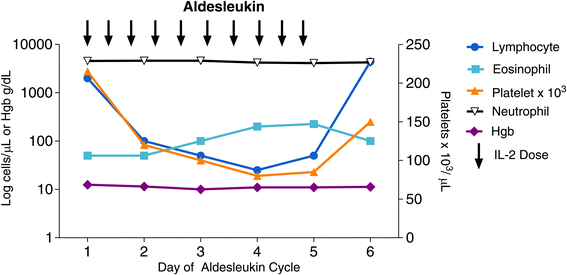

Supplement: Supplementary file 6 — Authors’ original file for figure 5 [file 40425_2014_26_MOESM6_ESM.gif]
